# A Rare Presentation of Polyarteritis Nodosa (PAN)

**DOI:** 10.7759/cureus.55143

**Published:** 2024-02-28

**Authors:** Joud Enabi, Kejal Shah, Hema Kondakindi, Srikanth Mukkera

**Affiliations:** 1 Internal Medicine, Texas Tech University Health Sciences Center, Odessa, USA; 2 Rheumatology, Texas Tech University Health Science Center at Permian Basin, Odessa, USA

**Keywords:** pan, autoimmune, aneurysm, rheumatology, polyarteritis nodosom

## Abstract

Polyarteritis nodosa (PAN) is a connective tissue disease that affects arteries, causing necrotizing inflammation that can weaken the arterial walls, dilatation into aneurysms, and rupture in some cases. We present a case of a male with acute abdomen from aneurysmal rupture. The 48-year-old patient with a history of polysubstance use including cocaine and methamphetamines was admitted for acute hypoxic respiratory failure secondary to coronavirus disease 2019 (COVID-19) pneumonia and treated with broad-spectrum antibiotics and steroids. He also reported generalized abdominal pain and discomfort, and examination revealed abdominal distension that was diffusely tender on palpation, bowel sounds intact. Laboratory workup showed a progressive drop in hemoglobin requiring blood transfusions, no coagulopathy, anion gap metabolic acidosis, and lactic acidosis. Abdominal CT showed a 2 cm lobulated saccular aneurysm involving either the left gastric artery or splenic artery, associated with an extensive moderate amount of hemoperitoneum with hematomas (largest measuring up to 8.6 cm) abutting the gastric fundus and greater curvature of the stomach, which was likely secondary to aneurysmal rupture. Additionally, several other mesenteric vessels displayed some degree of dilation. Interventional radiology (IR)-guided splenic artery embolization for splenic artery aneurysm was done, after which his hemoglobin remained stable. The patient was given vaccine recommendations since splenic artery embolization would lead to asplenia. The aneurysms were attributed to either cocaine-related aneurysms or polyarteritis nodosa with visceral artery aneurysms. He denied rashes, oral ulcers, joint pain, subcutaneous nodules, blood in the urine, history of hepatitis or syphilis. Tertiary syphilis was ruled out after the Venereal Disease Research Laboratory (VDRL) test and rapid plasma reagin (RPR) test were negative. Complement C3 and C4 levels were normal. He was treated with high-dose IV methylprednisone after infection was ruled out. Due to the severity of PAN, therapy with IV cyclophosphamide therapy 15 mg/kg once every two weeks for three doses was initiated, followed by 15 mg/kg once every three weeks for three to six months (in combination with glucocorticoids prednisone 1 mg/kg body weight with slow taper). Cyclophosphamide was given with IV hydration and mesna. The presentation of PAN can vary widely. Most commonly, individuals experience symptoms such as fatigue, weight loss, fever, and chills. However, in rare cases, patients may present with isolated abdominal pain, similar to our patient. It's crucial to note that the rupture of an aneurysm can manifest as an acute abdominal issue, potentially leading to life-threatening situations. Immediate interventions to control bleeding are imperative in such cases. The treatment of PAN has a high success rate when a combination of cyclophosphamide and steroids is administered.

## Introduction

Polyarteritis nodosa (PAN) is a connective tissue disease that predominantly affects medium-sized arteries and less commonly, small-sized arteries. It is an inflammatory vasculitis causing focal, segmental transmural necrotizing inflammation, that can weaken the arterial walls, causing dilatation into aneurysms.

PAN, a rare disease, has a worldwide incidence of 0.7 per 100,000 and a prevalence of 6.3 in the United States. Its exact pathology remains elusive, though it is believed to stem from an immune-mediated process affecting small to medium-sized blood vessels. Chronic inflammation of the elastic lamina in these vessels leads to necrosis and weakening of the vessel walls, a characteristic observed in arteriography in approximately 94% of PAN cases [1].

PAN presents with varied symptoms, including fatigue, weight loss, fever, chills, or, in rare instances, isolated abdominal pain [2]. A ruptured aneurysm can lead to an acute abdomen, a potentially life-threatening condition necessitating immediate intervention to control bleeding. Treatment for PAN typically involves a combination of cyclophosphamide and steroids, with high success rates [3].

## Case presentation

A 48-year-old male with a medical history of polysubstance use, including cocaine and methamphetamines, was transferred to our hospital from another facility where he presented with complaints of generalized abdominal pain that was insidious in onset and gradually progressive for the past three days, throbbing type, present intermittently, associated with nausea. He did not have any other medical history and did not take any medications at home. 

Prior to his transfer to our hospital, he had routine blood work done and tested positive for coronavirus disease 2019 (COVID-19) on polymerase chain reaction (PCR) and computerized tomography (CT) of the abdomen which showed soft tissue heterogeneous mass arising from the greater curvature and antrum of stomach measuring 6.5 x 13.2 x 8.6 cm, another heterogeneous density mass seen arising from the posterior wall of stomach measuring 10.5 x 8 x 9.7 cm with diffuse thickening and fat stranding, minimal ascites, and perihepatic space, ground glass attenuation in both lower lung lobes, emphysematous changes in both upper lobes, and minimal bilateral pleural effusion. He was treated with isotonic fluids, bronchodilators, empiric antibiotics for superimposed bacterial pneumonia, steroids, and analgesics prior to transfer. 

On arrival at our facility, he continued to be writhing in pain with a Glasgow Coma Score (GCS) of 15/15, afebrile with a temperature of 38.42 ^o^C, heart rate of 105 beats per minute, respiratory rate of 30 beats per minute, blood pressure 90/60 mmHg, and oxygen saturation (SPO2) at 98% on 2 L of oxygen through a nasal cannula. He required pressor support with norepinephrine to maintain a mean arterial pressure (MAP) goal of 65 mmHg. Blood work showed a normal white count and platelet count, hemoglobin of 8.5 g/dL (reference range: 13.0-17.7 g/dL), and normocytic and normochromic anemia. A complete metabolic panel showed acute kidney injury with creatinine 2.3 mg/dL (reference range: 0.5-1.4 mg/dl), no coagulopathy, elevated venous lactate of 6.3 with a normal anion gap, and inflammatory markers were mildly elevated including procalcitonin 1.31 ng/mL, D-dimer 3.32 mcg/mL, erythrocyte sedimentation rate (ESR) 24 mm/hour and C-reactive protein (CRP) 5.9 mg/dL. Chest x-ray showed bilateral infiltrates in the lower lobes with small pleural effusions bilaterally.

**Figure 1:**
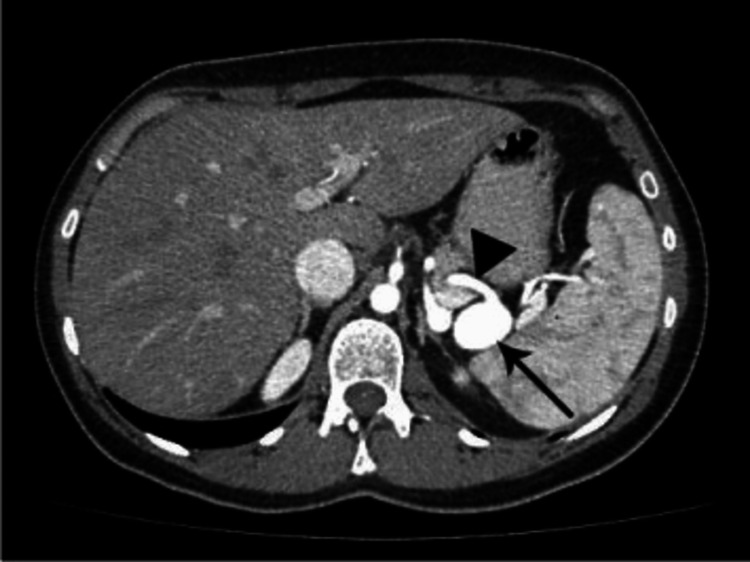
Contrast-enhanced CT scanning demonstrating the saccular aneurysm of the splenic artery (arrow).

He was initially treated for severe sepsis with shock secondary to pneumonia. However, he had a progressive drop in hemoglobin down to 6.3 mg/dL requiring blood transfusions, which prompted repeat imaging of the abdomen. A CT of the abdomen at our hospital showed a 2 cm lobulated saccular aneurysm involving the left gastric artery or splenic artery with a moderate amount of hemoperitoneum with hematomas (the largest measuring up to 8.6 cm) abutting the gastric fundus and greater curvature of the stomach which were likely due to aneurysmal rupture (Figure 1).

Additionally, there are several other mesenteric vessels, notably the superior mesenteric artery that displayed some degree of fusiform dilation. The interventional radiology (IR) team was consulted. Celiac and splenic arteriograms demonstrated a mid-splenic artery pseudoaneurysm. IR-guided splenic artery embolization with coils for the pseudoaneurysm was done, after which his hemoglobin remained stable and there were no further bleeds. 

The aneurysms were attributed to either cocaine or polyarteritis nodosa with visceral artery aneurysms. Tertiary syphilis was ruled out after the Venereal Disease Research Laboratory (VDRL) test and rapid plasma reagin (RPR) test were negative. A rheumatology consult was obtained. On further review, he denied rashes, oral ulcers, joint pain, subcutaneous nodules, blood in the urine, history of hepatitis or syphilis. PAN is frequently caused by the hepatitis B or C virus [4], but the patient’s hepatitis panel was negative. Cytoplasmic and perinuclear antineutrophil cytoplasmic antibodies (ANCAs) were negative. Given his elevated blood urea nitrogen and creatinine, characteristic imaging findings, and overall clinical picture, we diagnosed him with PAN, although the patient did not fulfill the American College of Rheumatology (ACR) classification criteria for PAN [5].

He was treated with high dose IV methyl prednisone for PAN, after infection was ruled out. Due to the severity, therapy with IV cyclophosphamide therapy 15 mg/kg once every two weeks for three doses was initiated, followed by 15 mg/kg once every three weeks for three to six months, in combination with a glucocorticoid taper with prednisone. Cyclophosphamide was given with IV hydration and mesna. 

## Discussion

In this patient, with his presenting symptoms and the results of his imaging studies, the aneurysms in multiple arteries, including splenic, gastric, and superior mesenteric arteries, were attributed to connective tissue disease causing vasculitis. Although obtaining a biopsy from the aneurysm was challenging to confirm the diagnosis of PAN conclusively, it fit the overall clinical picture of our patient.

Among the various classes of vasculitis diseases, the primary type to consider in this patient will be vasculitis involving the medium and large arteries in adults. Aside from PAN, diseases that match these measures include mycotic aneurysms, Takayasu arteritis, and cocaine-induced aneurysms. Analyzing the various possible etiologies of vascular aneurysms in this patient, PAN is the most plausible explanation. In this case, although the patient did present with a clinical picture of sepsis, his blood cultures and syphilis VDRL and RPR test were negative, which makes mycotic aneurysm unlikely. Takayasu arteritis was less likely in this patient as this vasculitis has a low rate of incidence, 2.6-6.4 cases per 1,000,000 population, affecting mainly the southeastern Asian population, with females affected more than males (80-90% female) [6,7]. Cocaine-related vascular aneurysms mainly affect cranial blood vessels and contribute to many subarachnoid hemorrhage cases [8].

The most common risk factors associated with splenic artery aneurysms include liver cirrhosis, portal hypertension, pregnancy, fibromyalgia, and congenital abnormalities. This case is unique and interesting, in that the patient also had a splenic artery aneurysm associated with PAN that ruptured and could have potentially been fatal. Splenic artery aneurysmal rupture can present as an acute abdomen, as in our patient, with abdominal pain, nausea, vomiting and associated hypotension, tachycardia, and syncopal episodes, causing hemorrhagic shock. Although, angiography is the standard for diagnosing aneurysms, CT and magnetic resonance imaging (MRI) scans can also be used for further imaging [9]. Splenic artery aneurysms are managed based on severity, size, and location. In patients who present with aneurysmal rupture, surgical interventions like excision, ligation, and revascularization, with or without splenectomy are beneficial [10]. Endovascular techniques, particularly coil embolization, are minimally invasive and can be considered when appropriate, or elective settings, and for those patients who are not surgical candidates.

Polyarteritis nodosa is a connective tissue disease that affects the arteries and causes necrotizing inflammation that can weaken the arterial walls, dilatation into aneurysms, and rupture in some cases. As a result, the organs supplied by the affected blood vessels suffer from poor blood perfusion that can cause organ infarction and ischemic atrophy consequently [3]. The exact underlying pathology of PAN still needs to be better understood. However, it is believed to be a result of an immune-mediated process affecting blood small to medium blood vessels [11]. Polyarteritis nodosa is a rare disease with an incidence of 0.7 in 100,000 worldwide and a prevalence in the United States of 6.3 in 100,000 [2,12]. PAN has a high incidence in patients aged 40-60 years and primarily affects men with a male-to-female ratio of 1.6:1, with no known geographical distribution [2,12]. Patients with PAN have a variable presentation, from mild skin lesions to systemic fulminant variety. PAN mainly causes fatigue, weight loss, fever, and chills in up to 70% of the patients [13]. On the other hand, 65% of cases may present with polyneuropathy and mononeuritis multiplex. Furthermore, isolated abdominal pain, as in our patient, can be seen in around 40% of the reported PAN cases [14].

Non-specific inflammatory findings can be present in the laboratory tests of patients with PAN, including normocytic normochromic anemia, leukocytosis, elevated ESR, and proteinuria. Furthermore, a low percentage of patients with PAN, up to 20%, can test positive for antineutrophil cytoplasmic antibodies (ANCA). On the other hand, 25% of patients have suppressed complement levels C3 and C4 [15]. The gold standard test to diagnose PAN is arteriography, with a characteristic finding of diffused aneurysms that mainly affect the arteries in the kidneys and liver. The aneurysms result from chronic inflammation of the elastic lamina of the small and medium arteries, resulting in necrosis and weakness of the vessel walls, which can be found in the arteriography of around 94% of PAN cases [16], as in our patient. Corticosteroids are the primary treatment used to treat PAN initially. However, response to the steroid regimen can be determined within the first three months of treatment [15]. Patients who respond well to steroid treatment require a prolonged course of treatment to keep the disease in remission. On the other hand, cyclophosphamide is considered an alternative treatment option for those who cannot tolerate corticosteroids or had a poor response to the treatment [13]. Studies have shown improvement in patients' survival after being put on combination therapy with steroids and cyclophosphamide when compared to treatment with steroids alone [16].

## Conclusions

PAN is a connective tissue disorder primarily impacting medium-sized arteries and, to a lesser extent, small arteries. It represents an inflammatory vasculitis characterized by focal, segmental transmural necrotizing inflammation, which may lead to arterial wall weakening and subsequent formation of aneurysms. PAN patients exhibit a spectrum of presentations, ranging from minor skin manifestations to severe systemic involvement. While occurrences of splenic artery aneurysms in PAN are uncommon, their rupture represents a rare yet highly dangerous complication.
